# Supplementary material for: Effectiveness and Safety of Ayurvedic Medicines in Type 2 Diabetes Mellitus Management: A Systematic Review and Meta-Analysis
Source: Front Pharmacol. 2022 Jun 8;13:821810. doi: 10.3389/fphar.2022.821810 (PMC9213670; doi:10.3389/fphar.2022.821810)
Supplement: Supplementary file 1 [file DataSheet1.zip › Supplementary Material/Forest and Funnel Plots/Portulaca oleracea/TG.pdf]

Portulaca oleracea - TG

	Intervention			Comparator				
Study	N	Mean	SD	N	Mean	SD	Mean Diff. with 95% CI	Weight (%)
Farzanegi 2014	14	134.29	33.40	14	156.72	46.74	-22.43 [-52.52, 7.66]	44.92
Dehghan 2016	16	150.75	60.09	16	167.80	75.53	-17.05 [-64.34, 30.24]	32.74
Wainstein 2016	31	206.40	169.60	32	150.70	92.90	55.70 [-11.54, 122.94]	22.34
**Overall**							-3.21 [-43.31, 36.88]	

Heterogeneity: $\tau^2 = 696.01$, $I^2 = 55.80\%$, $H^2 = 2.26$

Test of $\theta_i = \theta_j$: Q(2) = 4.40, p = 0.11

Test of $\theta = 0$: z = -0.16, p = 0.88

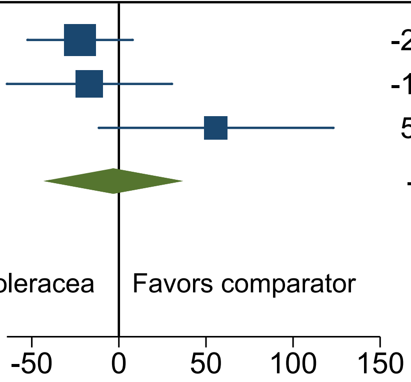

Random-effects REML model
